# Mutations of *KRAS/NRAS/BRAF* predict cetuximab resistance in metastatic colorectal cancer patients

**DOI:** 10.18632/oncotarget.8076

**Published:** 2016-03-12

**Authors:** Hung-Chih Hsu, Tan Kien Thiam, Yen-Jung Lu, Chien Yuh Yeh, Wen-Sy Tsai, Jeng Fu You, Hsin Yuan Hung, Chi-Neu Tsai, An Hsu, Hua-Chien Chen, Shu-Jen Chen, Tsai-Sheng Yang

**Affiliations:** ^1^ Division of Hematology-Oncology, Chang Gung Memorial Hospital, Kwei-Shan, Tao-Yuan 333, Taiwan; ^2^ College of Medicine, Chang Gung University, Kwei-Shan, Tao-Yuan 333, Taiwan; ^3^ ACT Genomics, Neihu Dist., Taipei 114, Taiwan; ^4^ Division of Colon and Rectal Surgery, Chang Gung Memorial Hospital, Kwei-Shan, Tao-Yuan 333, Taiwan; ^5^ Graduate Institute of Clinical Medicine, College of Medicine, Chang Gung University, Kwei-Shan, Tao-Yuan 333, Taiwan

**Keywords:** BRAF, metastatic colorectal cancer, RAS, mutation, cetuximab resistance

## Abstract

Approximately 45% of metastatic colorectal cancer (mCRC) patients with wild-type KRAS exon 2 are resistant to cetuximab treatment. We set out to identify additional genetic markers that might predict the response to cetuximab treatment. Fifty-three wild-type KRAS exon 2 mCRC patients were treated with cetuximab/irinotecan-based chemotherapy as a first- or third-line therapy. The mutational statuses of 10 EGFR pathway genes were analyzed in primary tumors using next-generation sequencing. *BRAF*, *PIK3CA*, *KRAS* (exons 3 and 4), *NRAS*, *PTEN*, and *AKT1* mutations were detected in 6, 6, 5, 4, 1, and 1 patient, respectively. Four of the *BRAF* mutations were non-V600 variants. Four tumors harbored multiple co-existing (complex) mutations. All patients with *BRAF* mutations or complex mutation patterns were cetuximab non-responders. All patients but one harboring *KRAS*, *NRAS*, or *BRAF* mutations were non-responders. Mutations in any one of these three genes were associated with a poor response rate (7.1%) and reduced survival (PFS = 8.0 months) compared to wild-type patients (74.4% and 11.6 months). Our data suggest that *KRAS, NRAS*, and *BRAF* mutations predict response to cetuximab treatment in mCRC patients.

## INTRODUCTION

Despite recent advances in treatment, nearly 600,000 colorectal cancer- (CRC) related deaths occur annually [1], and CRC is the most common cancer in Taiwanese patients. Many patients with advanced CRC experience recurrence after surgical resection, the primary treatment for this disease. Furthermore, > 25% of CRC patients have liver metastases at the time of initial diagnosis, and approximately 50% of patients eventually develop metastases. The 5-year survival rate is as low as 10~20% for patients with distant metastatic disease [2]. While screening, surgery, and medical therapies are effective in the management of early-stage CRC, these treatment options are far less efficacious in advanced stages. Inter- and intratumoral genetic heterogeneity is key factor in predicting treatment failure and drug resistance in CRC therapies [3–4].

In the past decade, targeted biologic therapies, including monoclonal antibodies targeting vascular endothelial growth factor (VEGF) and epidermal growth factor receptor (EGFR), have significantly improved clinical outcomes in patients with CRC [5]. Ligand-induced EGFR activation in particular plays a pivotal role in tumor proliferation, invasion, migration, and neovascularization through the RAS-RAF-MAPK and PI3K-AKT-mTOR pathways [6]. The EGFR-targeting antibodies cetuximab, a chimeric IgG1 monoclonal antibody, and panitumumab, a humanized IgG2 monoclonal antibody, have proven effective against CRC in clinical trials and have been used either individually or in combination with standard chemotherapy to improve survival in metastatic CRC (mCRC) patients [7–8]. However, mutations in effector signaling molecules downstream of EGFR activate receptor-independent pathway (s) that render tumors unresponsive to EGFR inhibition treatment. Randomized phase III studies provide compelling evidence that the EGFR-targeting monoclonal antibodies cetuximab and panitumumab are effective only in CRC patients harboring wild-type *KRAS* exon 2 [9–10]. Consequently, regulatory authorities have mandated the implementation of *KRAS* exon 2 mutation screening when selecting patients for anti-EGFR treatment [8, 11].

Although the *KRAS* exon 2 mutation has been established as an important biomarker for predicting responsiveness to anti-EGFR treatment, approximately 40~50% patients harboring wild-type *KRAS* exon 2 do not benefit from these targeted agents, suggesting the potential involvement of genetic alterations in the KRAS/BRAF and PI3K/AKT pathways downstream of EGFR. All of these genes have been associated with tumor growth and progression, and recent studies suggest that additional mutations in *KRAS* and *NRAS*, as well as downstream mutations in *BRAF* or *PIK3CA*, may cause resistance to anti-EGFR treatment [12]. However, these findings were based on clinical studies containing patients of different races in which few genes were analyzed, and the results were often contradictory [13]. Previous studies have found higher rates of *KRAS* mutations in Asian patients with small-cell lung carcinoma, reflecting an ethnic difference in cancer genomics [14]. However, factors influencing *KRAS* and other effectors downstream of EGFR in CRC specifically in Asian populations have not been systemically evaluated, particularly in the Taiwanese population. Additional molecular studies identifying other predictive biomarkers may help to optimize anti-EGFR therapies in mCRC patients.

Since the launch of the first massively parallel sequencing platform in 2005, next-generation sequencing (NGS) technologies have evolved rapidly, revolutionizing the scale of genomic studies and providing powerful diagnostic tools for implementing precision medicine through high-throughput genomic analysis [15]. As NGS techniques continue to improve and decrease in cost, it has become feasible to routinely employ NGS in clinical settings to analyze large-scale genetic information regarding inter- and intratumoral gene alterations, allowing better stratification of patients when selecting personalized therapies. In the present study, we utilized NGS technology to analyze the EGFR signaling pathway genes *EGFR, KRAS, HRAS, NRAS, BRAF, PIK3CA, AKT1, PTEN, HER2*, and *HER4* in a wild-type *KRAS* exon 2 cohort of 53 Taiwanese mCRC patients undergoing cetuximab treatment. Our results demonstrate that poor responses to cetuximab in CRC patients can be attributed to a combinational set of gene mutations in addition to mutations in *KRAS* exon 2.

## RESULTS

### Patient characteristics

A total of 53 mCRC patients were treated with cetuximab in combination with chemotherapy either as a first line (*n =* 39, 73.5%) or third line (*n =* 14, 26.4%) therapy. All subjects were confirmed as having wild-type *KRAS* exon 2 before cetiximab administration. Follow-ups were conducted with each patient every week to three months until March 1, 2014, or until death. The mean follow-up duration was 17.1 months, with a standard deviation of 10.1 months. Patient age at diagnosis ranged from 28 to 93 years (mean, 63.5 ± 14.0). The tumor subsites were the colon (32 patients) and rectum (21 patients). Tumors were staged according to the AJCC 2010 guidelines. The main characteristics of all enrolled patients are summarized in Table 1.

**Table 1:** Characteristics of patients with metastatic colorectal cancer

	All patients	Responders	Non-responders	*p*-value^*^
	*N* (%)	*N* (%)	*N* (%)	
Total number	53 (100)	30 (56.6)	23 (43.4)	
Sex				0.775
Male	34 (64.2)	20 (66.7)	14 (60.8)	
Female	19 (35.8)	10 (33.3)	9 (39.2)	
Age				0.052
≤ 70	40 (75.5)	26 (86.7)	14 (60.9)	
> 70	13 (24.5)	4 (13.3)	9 (39.1)	
Median (range)	57 (32–83)	53.5 (32–83)	61 (36–79)	
Histologic Grade				0.222
low grade^1^	47 (60)	28 (63.3)	19 (56.5)	
high grade^2^	6 (40)	2 (36.7)	4 (43.5)	
Metastatic pattern				0.375
metachronous	17 (66)	9 (72.9)	8 (57.1)	
synchronous	36 (34)	21 (27.1)	15 (42.9)	
Primary Tumor site				0.778
Colon	32 (60.4)	19 (63.3)	13 (56.5)	
Rectum	21 (39.6)	11 (36.7)	10 (43.5)	
Metastatic site				
Liver	38	22	16	0.962
Lung	19	10	9	0.663
Other	22	8	14	0.012
Number of metastatic site				0.052
1	31	21	10	
> 1	22	9	13	
Treatment regimen				0.346
1st line	39 (74)	24 (80.0)	15 (65.2)	
3rd line	14 (26)	6 (20.0)	8 (34.8)	
PFS (months)				0.061
Median (range)	10.6 (2.7–51.7)	11.4 (4.4–51.7)	8 (2.7–26.4)	

* Fisher exact *p*-value.

1 well-differentiated/moderately-differentiated.

2 poorly-differentiated.

The cohort was separated into two groups based on patients' responses to cetuximab. The “responder” group included 30 (61.5%) patients with either complete remission (*n =* 2, 3.8%) or partial remission (*n =* 28, 52.8%). The “non-responder” group included 23 (43.4%) patients with stable disease (*n =* 18, 34%) or progression (*n =* 5, 9.4%). Response rates were similar among the first-line subjects (61.5%, 24 of 39) and the third-line subjects (42.9%, 6 of 14) (*p* = 0.346). Median response durations were also similar among first-line subjects (10.83 months) and third-line subjects (11.6 months) (*p* = 0.370).

### Targeted sequencing of genes involved in EGFR signaling pathway

In order to identify genetic alterations in the EGFR signaling pathway that affect cetuximab treatment responses, we applied NGS technology to analyze frequently mutated regions of 10 EGFR-related genes, including *EGFR, KRAS, HRAS, NRAS, BRAF, PIK3CA, AKT1, PTEN, HER2*, and *HER4* (targeted regions listed in Supplementary Table S1). The *TP53* gene, one of the most frequently mutated genes in CRC, was sequenced as a control. All samples were sequenced at an average depth of > 1000 × (Supplementary Table S2). Raw sequence data were aligned to a human reference genome to identify variants. After annotation, variants were filtered to remove single nucleotide polymorphisms (SNPs) and synonymous mutations. Only the non-synonymous mutations were used for subsequent analysis.

**Figure 1:**
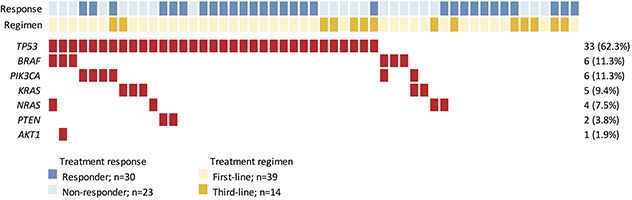
Heat-map representation of individual non-silent variants identified in a total of 53 specimens Light blue and dark blue highlighting indicates individual responses to cetuximab-based treatment. Light and dark yellow highlighting indicates which cetuximab-based regimen each individual received. Individuals highlighted in red had non-silent mutations. The columns in the Table denote the samples, and the rows denote the genes. The right panel indicates the mutation frequency of each gene in the cohort.

We detected 61 non-synonymous variants in 7 genes, including *AKT1, BRAF, KRAS, NRAS, PIK3CA, PTEN*, and *TP53* (Figure 1, Supplementary Table S3), in 40 subjects. As expected, *TP53* was the most frequently mutated gene detected in the cohort. Thirty-three subjects exhibited mutations in the *TP53* gene, resulting in a population frequency of 62.3%. The second most frequently altered genes were *BRAF* and *PIK3CA*, with mutations detected in 6 subjects (11.3%) for each. The third most frequently mutated gene was *KRAS*, with a 9.4% (*n =* 5) population frequency, followed by *NRAS* (*n =* 4, 7.5%), *PTEN* (*n =* 2, 3.7%) and *AKT1* (*n =* 1, 1.9%). The mutation frequencies of *TP53*, *BRAF*, and *NRAS*, but not *KRAS*, detected in this study are very similar to those found by Brannon et al. [16–17].

Mutations in EGFR-related genes were detected in 20 (38%) subjects (Figure 1). Sixteen subjects harbored a single mutation while 4 subjects displayed a complex mutation pattern with multiple mutations co-existing in the same tumor. Interestingly, 3 of 6 subjects with *BRAF* mutations displayed complex mutation patterns (*BRAF*+*AKT1*, *BRAF*+*PIK3CA*, *BRAF*+*NRAS*). Two complex mutation patterns were observed for *PIK3CA* mutations (*PIK3CA*+*BRAF*, *PIK3CA*+*KRAS*). These results indicate that, while *KRAS* and *NRAS* tend to exhibit mutually exclusive mutation patterns, mutations in signaling molecules downstream of EGFR may co-exist with *KRAS* and *NRAS* alterations in colorectal tumors.

### Genetic alterations are more common in cetuximab non-responders

**Figure 2:**
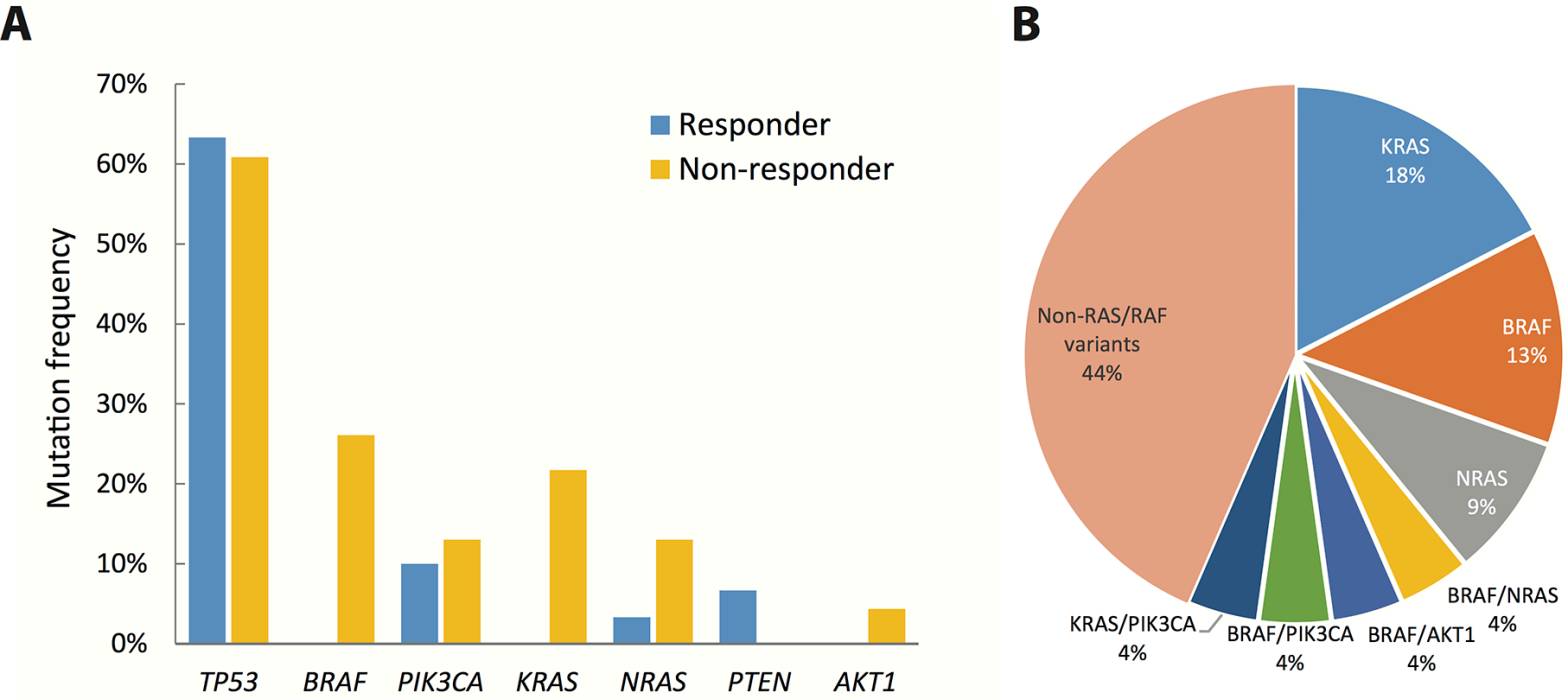
Extent of genetic disruption in metastatic CRC (**A**) Prevalence of tumors harboring non-silent mutations in cetruximab responders (blue) and non-responders (yellow). (**B**) Distribution of genetic disruptions in the cetuximab non-responder group.

We next compared the mutation frequencies of individual genes between cetuximab responders and non-responders. As shown in Figure 2A and Table 2, mutation frequencies of *TP53* (63.3% for responders vs. 60.9% for non-responders, Fisher's exact test: *p =* 1.000) and *PIK3CA* (10% for responders vs. 13% for non-responders,*p =* 1.000) were similar between responders and non-responders. In contrast, *BRAF* mutations (0% for responders vs. 26.1% for non-responders, *p =* 0.0044) and *KRAS* (0% for responders vs. 21.7% for non-responders, *p =* 0.0117) were observed exclusively in non-responders. Three of the 4 subjects with *NRAS* mutations were non-responders (*p =* 0.3053) while both *PTEN* mutations were detected in responders (*p =* 0.4993). Collective mutation frequencies of *KRAS, NRAS*, and *BRAF* combined were higher in non-responders (*p =* 0.0001).

**Table 2:** Association between genetic alterations and treatment outcome

Gene symbol	Mutation status	Treatment outcome		*p* value^*^
		Responders (*n* = 30)	Non-responders (*n* = 23)	
*BRAF*	WT	30	17	0.004
	Mut	0	6	
*KRAS*	WT	30	18	0.012
	Mut	0	5	
*NRAS*	WT	29	20	0.305
	Mut	1	3	
*BRAF/KRAS/NRAS*	WT	29	10	0.000
	Mut	1	13	
*PIK3CA*	WT	27	20	1.000
	Mut	3	3	
*AKT1*	WT	30	22	0.434
	Mut	0	1	
*PTEN*	WT	28	23	0.499
	Mut	2	0	
*PIK3CA/AKT1/PTEN*	WT	25	19	1.000
	Mut	5	4	
*TP53*	WT	11	9	1.000
	Mut	19	14	

* Fisher exact *p*-value

Among the 23 non-responders, 4 (17.4%) subjects had a non-exon 2 *KRAS* mutation, 3 (13%) subjects had a *BRAF* mutation, and 2 subjects (8.7%) had an *NRAS* mutation (Figure 2B). In addition to single mutations, 4 subjects showed complex mutations in both the *KRAS/PIK3CA*, *BRAF/NRAS*, *BRAF/AKT1*, and *BRAF/PIK3CA* genes. Collectively, genetic alterations in *BRAF*, *NRAS* and *KRAS* were detected in 13 (56.5%) of the non-responders. These genetic alterations may contribute to resistance to cetuximab; the mechanism of cetuximab resistance for the remaining 10 (43.5%) subjects requires further investigation.

### Genetic alterations related to cetuximab resistance

**Figure 3:**
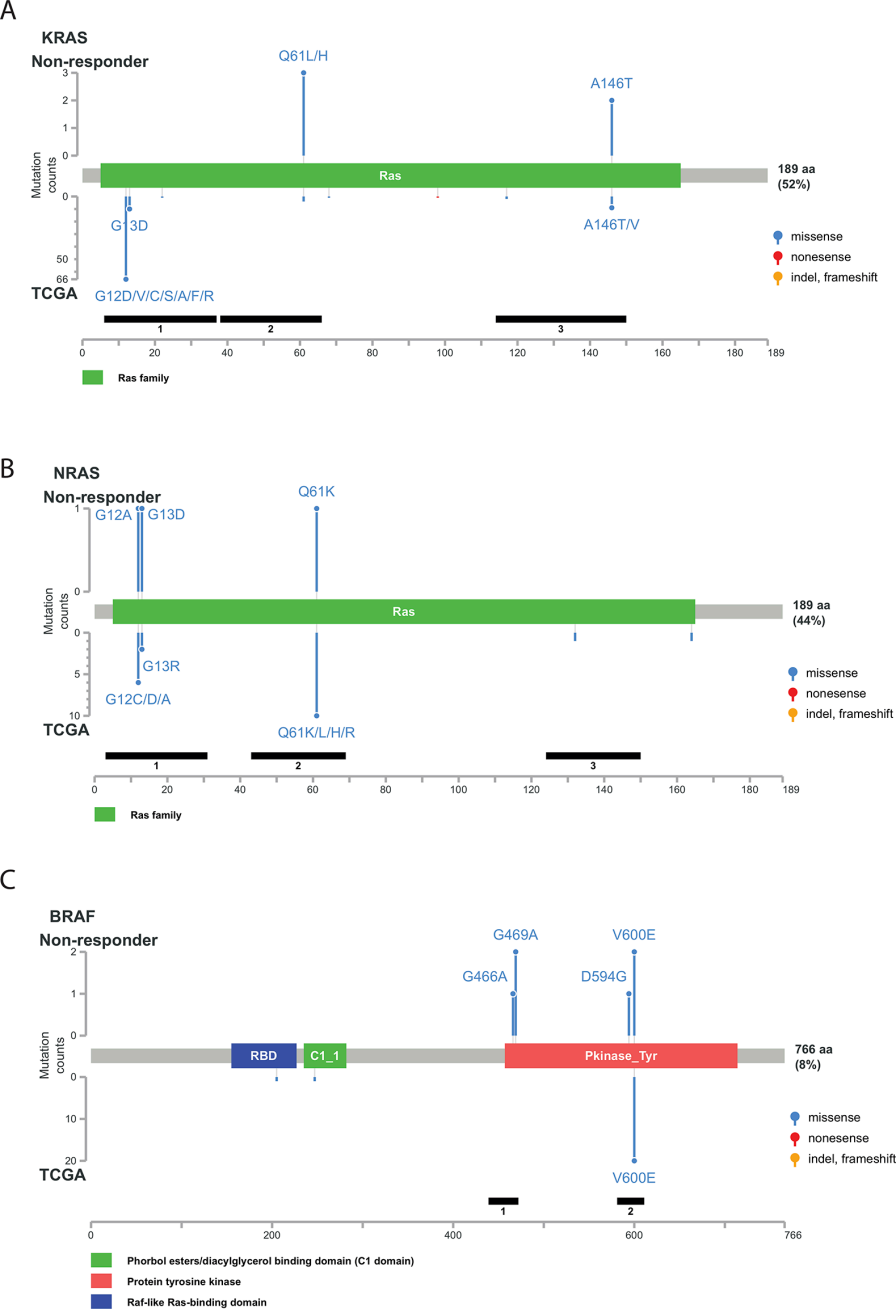
Distribution of genetic alterations detected in the non-responder group Schematic representation of protein structures of KRAS (**A**) NRAS (**B**) and BRAF (**C**). The upper panel indicates the genetic alterations detected in the non-responder group. The lower panel indicates the mutation spectrum in The Cancer Genome Atlas (TCGA) colorectal cancer dataset. The left scale indicates the number of cases. The blue, yellow, red, and purple circles indicate missense mutations, insertions/deletions, nonsense mutations, and multiple types of mutations, respectively.

To further explore the relationship between genetic alterations and cetuximab resistance, we analyzed the *KRAS*, *NRAS*, and *BRAF* mutant alleles detected in non-responders and compared mutation patterns of the individual genes to data published by the Cancer Genome Atlas (TCGA) Research Network [17]. Activating mutations in a few well-characterized loci were detected in the *KRAS* and *NRAS* genes (Figure 3A, 3B). For example, two Q61L (exon 3), one Q61H (exon 3), and one A146T (exon 4) *KRAS* mutations were detected in the non-responders. All of these rare mutations activate KRAS activity [18–19]. These loci are not included in the routine *KRAS* exon 2 test but are frequently mutated in CRC patients. Similarly, activating *NRAS* mutations (G12A, G13D and Q61K) are not included in routine tests for CRC patients before cetuximab administration. Mutations in both genes are frequently detected in CRC patients and are present at a higher frequency in cetuximab non-responders compared with responders. These additional *RAS* mutations were also verified in large randomized trials and are included in the all-RAS mutational test recently recommended by the National Comprehensive Cancer Network [20].

Among the *BRAF* mutations observed in CRC, a single nucleotide mutation in codon 600 of the kinase domain resulting in substitution of glutamic acid for valine (V600E) accounted for over 95%. In fact, the V600E allele was the only somatic mutation detected in the TCGA CRC samples [21]. In comparison, among the 6 subjects with *BRAF* mutations in this cohort, only 2 (33.3%) had *BRAF* V600E. Other genetic alterations in *BRAF* included G466A (1 subject), G469A (2 subjects) and D594G (1 subject) (Figure 3C). These three mutations also occurred in the protein kinase domain and were not recorded in the TCGA colorectal cancer dataset. Furthermore, previous studies suggest that *PIK3CA* exon 20 mutations, but not *PIK3CA* exon 9 mutations, are associated with cetuximab resistance in CRC [22]. *PIK3CA* mutations in exon 9 (helical domain) were detected in 5 subjects, and only 1 subject had a *PIK3CA* mutation in exon 20 (kinase domain) (Supplementary Figure S1). The high frequency of *PIK3CA* exon-9 mutations (E542K and E545K) was similar to that observed in Japanese CRC patients [23]. All *PIK3CA* mutations identified in this study have been reported in metastatic CRC before.

### Genetic alterations and treatment response

**Figure 4:**
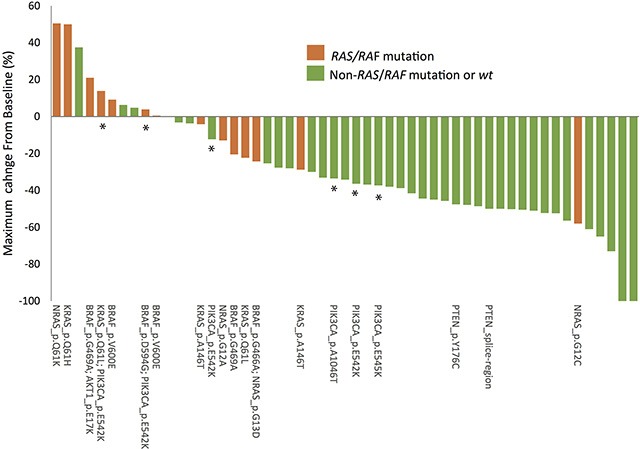
Relationship between responses to cetuximab-based treatment and genetic mutations The waterfall plot depicts the percentage change in tumor size from baseline 3 months after cetuximab-based treatment. *indicates samples with mutations in PIK3CA.

In the responder group, 2 patients showed complete response (CR) and 28 showed partial response (PR) for a 60.1% overall response rate (ORR) to cetuximab. In the non-responder group, 18 patients had stable disease (SD) and 5 had progressive disease (PD). The waterfall plot illustrated the relationship between genetic alterations and treatment responses (Figure 4). All patients with mutations in *KRAS* or *BRAF* failed to respond to cetuximab treatment, and three subjects with *NRAS* mutations did not respond to cetuximab treatment. One subject with a low *NRAS* G12C mutant allele frequency (5%) did show a 50% tumor reduction after cetuximab treatment. Overall, tumors harboring mutations in the KRAS-NRAS-BRAF axis responded poorly to cetuximab-based treatment. Responses to cetuximab-based treatment in tumors harboring mutations in the PTEN-PI3K-AKT signaling pathway were more complex. Both tumors harboring *PTEN* mutations responded well to the treatment. All four subjects with a single *PIK3CA* mutation showed weak to moderate tumor regression (−14%, −34%, −36%, and −37%).

In addition to single mutations, complex mutational patterns were frequently detected in mCRC patients. Notably, none of the tumors harboring complex mutations responded to cetuximab-based treatment. Only one tumor harboring complex *BRAF*/*NRAS* mutations showed weak tumor regression (−24%). The remaining three tumors with complex mutations showed disease progression (4%, 14% and 21% changes in tumor size) during cetuximab-based treatment. These results suggest that these complex mutational patterns also predict poorer response toward cetuximab treatment. We used a microsatellite instability (MSI) test to determine whether complex mutational patterns were associated with genetic hypermutability caused by impaired DNA mismatch repair (MMR). All samples with complex mutations showed the microsatellite stable (MSS) phenotype, suggesting that the concomitant mutations represent a complex perturbation of the RAS/RAF signaling pathway in advanced CRC rather than a consequence of MSI-associated hypermutation. Furthermore, the frequency of individual mutant alleles differed in some of the tumors harboring complex mutational patterns. For example, tumor A00021 had both *AKT1* E17K (27.9%) and *BRAF* G469A (19.7%) mutations, and tumor A00026 had both *BRAF* D594G (26.7%) and *PIK3CA* E542K (15.6%) mutations. Such differences in mutant allele frequencies implies complex intra-tumor heterogeneity, which may also contribute to the lack of response to cetuximab.

### Genetic alteration and duration of treatment response

**Figure 5:**
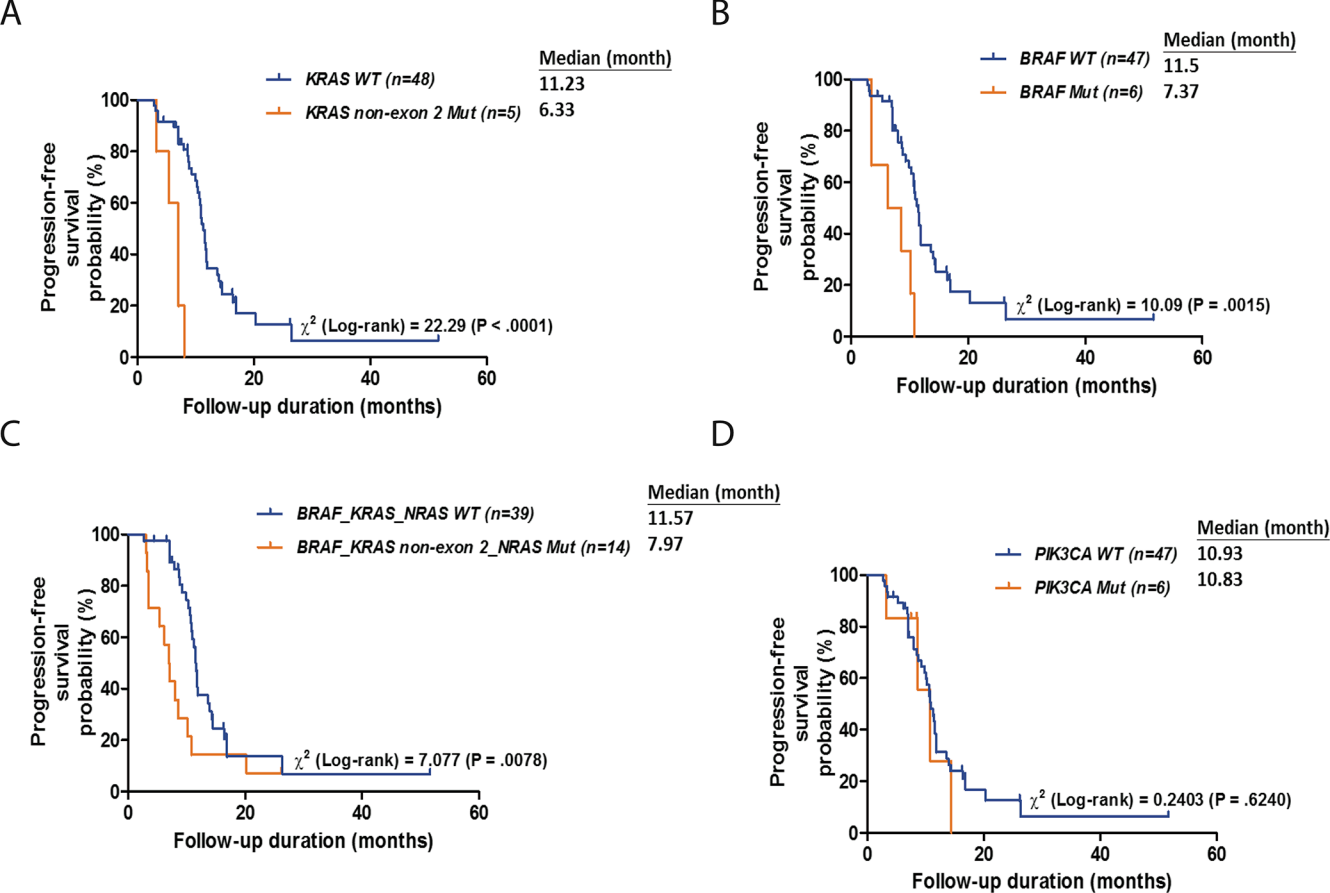
Kaplan-Meier plots for DSS according to the mutation status of KRAS (A), BRAF (B), BRAF, KRAS or NRAS (C) and PIK3CA (D) *p* values were calculated using the log-rank test.

In addition to changes in tumor size, we also examined whether genetic mutations affected the duration of response. The median duration of response to cetuximab-based treatment was 10.6 months (range, 2.7 to 51.7 months; mean, 11.4 months). The median response durations the 30 responders and the 23 non-responders were 11.4 months (range, 4.4 – 51.7 months; mean, 13.1 months) and 8 months (range, 2.7 to 26.4 months; mean, 9.1 months), respectively. A Kaplan-Meier plot revealed that patients with mutated *KRAS* exon 3/4 had shorter median response durations than patients with wild-type *KRAS* (6.3 vs. 11.2 months, respectively; log-rank *p*-value < 0.0001; Figure 5A). Similarly, patients with mutated *BRAF* had shorter median response durations than patients with wild-type *BRAF* (7.4 vs. 11.5 months, respectively; log-rank *p*-value = 0.0015; Figure 5B). Furthermore, patients with mutated *KRAS/NRAS/BRAF* had lower response rates and shorter median response durations than wild-type patients (7.1% vs. 74.4% and 11.6 vs. 8.0 months, respectively; log-rank *p*-value = 0.0078; Figure 5C). In contrast, no difference in response duration was observed between patients with mutated and wild-type *PIK3CA* genes (*p* = 0.624; Figure 5D).

We further evaluated the ability of genetic mutations and other clinicopathological risk factors, including sex (male vs female), age (> 70 vs ≤ 70), pT stage (T4 vs T2–3), pN stage (N2 vs N0–1), p-Stage (IV vs I–III), histologic grade (high vs low), metastatic pattern (metachronous vs synchronous), primary tumor site (rectum vs colon), number of metastatic sites (> 1 vs 1), and treatment regimen (3rd-line vs 1st-line), to predict cetuximab response duration. Univariate analysis revealed that higher age, multiple metastatic sites, *KRAS* mutations, *BRAF* mutations, and mutated *KRAS/NRAS/BRAF* were associated with shorter response durations (Table 3). Multivariate analysis indicated that the presence of multiple metastatic sites and mutated *KRAS*, *BRAF*, and *KRAS/NRAS/BRAF* were independent risk factors for poorer response (Table 3). Hazard ratios for patients with mutated *KRAS*, *BRAF*, or *KRAS/NRAS/BRAF*, compared to patients with wild-type genes, were 8.479 (95% CI = 2.524–28.487, *p =* 0.001), 2.603 (95% CI = 1.017–6.661, *p =* 0.046), and 2.716 (95% CI = 1.345–5.481, *p =* 0.005), respectively. These results further support that mutated *KRAS*, *BRAF*, and *KRAS/NRAS/BRAF* are independently associated with cetuximab response even after considering traditional risk factors.

**Table 3:** Univariate and multivariate analysis of factors associated with progression-free survival

	*n* = 53	Univariate analysis			Multivariate analysis		
Factors	No.	HR	95% CI	*p*-value	HR	95% CI	*p*-value
Clinical features							
Sex (Male/Female)	34/19	0.911	0.483–1.720	0.775			
Age (> 70/≤ 70)	13/40	2.113	1.079–4.138	0.029	1.926	0.964–3.846	0.063
pT stage (T4/T23)	22/31	1.621	0.870–3.017	0.128			
pN stage (N2/N01)	28/25	1.45	0.781–2.692	0.239			
Stage (stage4/stage1–3)	35/18	0.74	0.387–1.415	0.363			
Histologic Grade (high/low)	6/47	2.055	0.855–4.939	0.108			
Metastatic pattern (meta/syn)	17/36	1.247	0.646–2.406	0.510			
Primary Tumor site (rectum/colon)	21/32	0.936	0.504–1.737	0.834			
Number of metastatic sites (> 1/1)	22/31	3.625	1.847–7.112	< 0.001	3.668	1.828–7.360	< 0.001
Treatment regimen (3rd line/1st line)	14/39	0.725	0.357–1.472	0.373			
Genetic alteration							
KRAS (Mut/WT)	5/48	9.502	3.056–29.55	< 0.001	8.479	2.524–28.49	0.001
BRAF (Mut/WT)	6/47	4.009	1.583–10.15	0.003	2.603	1.017–6.661	0.046
NRAS (Mut/WT)	4/49	0.656	0.193–2.227	0.499			
KRAS/NRAS (Mut/WT)	9/44	1.779	0.795–3.982	0.161			
BRAF/KRAS/NRAS (Mut/WT)	14/39	2.447	1.239–4.832	0.010	2.716	1.345–5.481	0.005

## DISCUSSION

To date, *KRAS* exon 2 mutations are the most commonly used biomarker for predicting the therapeutic efficacy of anti-EGFR antibodies in advanced CRC. However, it has become clear that the *KRAS* exon 2 mutation alone is insufficient for predicting responsiveness to such therapies. The prognostic and predictive relevance of other genes in the RAS-RAF and PI3K-AKT-mTOR pathways are now being investigated in CRC patients with different ethnic backgrounds. However, the role of these genes is somewhat controversial, in part because previous studies include patients of various races, analyze only a few biomarkers, and employ diverse experimental methods. Here, we analyzed genes in the EGFR signaling pathway in a well-characterized Taiwanese cohort to complete the first study in Asian CRC patients undergoing first- or third-line cetuximab treatment in combination with chemotherapy. Our study clearly illustrates the predictive importance of mutations outside of the *KRAS* exon 2. Mutations in *KRAS* exons 3 and 4, *BRAF*, and *NRAS* served as strong predictors of PFS poor responses to EGFR-targeted therapies. More importantly, our data demonstrate that significantly higher predictive power might be achieved by analyzing combinations of mutations in these 3 genes, which occur in 80% of cetuximab non-responders.

In the past, multiplex mutation screening was challenging due to inadequate sample availability, poor tumor purity, difficulty in including rarer mutations, and technical obstacles. We overcame these technical problems and established a multiplex mutation assay for clinical CRC samples by exploiting two-step tagging PCR and NGS technology for targeted amplicon sequencing, allowing simultaneous detection of very low levels of point mutations in targeted genes. PCR-based enrichment coupled with the NGS procedure enabled us to perform high-sensitivity mutation profiling in tumor biopsy specimens. Applying the same approach may provide companion diagnostic tools for screening many other cancer-related genes in a multiplex, high throughput format.

The *BRAF* V600E substitution is a well-characterized oncogenic mutation in cancers such as melanoma. Previous studies found that *BRAF* mutations are confined to codon 600 in CRC tissues. In this study, we identified three additional mutations on exons 11 and 15 of the *BRAF* gene. Importantly, V600E mutations accounted for only one third of all *BRAF* substitutions in the Taiwanese cohort. In addition, 11% of mCRC patients in this cohort carried *BRAF* mutations, which is a larger proportion than observed previously in Caucasian populations. Our data illustrate that *BRAF* mutation types and frequencies may vary depending on the ethnic background, gender, and disease severity of patients. *BRAF* is strongly associated with metastasis, and *BRAF* mutations along with increased kinase activity usually upregulate MAPK cascade signaling [24]. Our results unequivocally indicate that various *BRAF* mutations were associated with shorter PFS in non-responders, suggesting that *BRAF* mutations may help identify those who would benefit from anti-EGFR treatment among Taiwanese patients with wild-type *KRAS*. Although the predictive value of *BRAF* mutations has been controversial in targeted therapies for mCRC [25–27], our study and many others demonstrate the utility of *BRAF*. For example, a meta-analysis by Wang *et al.* suggested that genetic aberrations in *BRAF* impair the efficacy of anti-EGFR therapy in Asian mCRC populations (4). A recent study by Nicolantonio *et al.* also found that *BRAF* mutations were associated with unfavorable clinical outcomes for cetuximab- or panitumumab-based therapies [28]. Further cell-based functional studies would clarify the significance of each *BRAF* mutant allele that contributes to CRC carcinogenesis.

PIK3CA kinase and PTEN phosphatase are potent regulators in the PI3K-AKT-mTOR pathway, and mutations in these genes have long been implicated in various tumor types, including breast, colorectal, endometrial, ovary, liver, and gastric malignancies, despite low incidences [29]. Nevertheless, there are conflicting reports regarding the association between *PIK3CA* mutations and resistance to cetuximab and panitumumab therapies in CRC [30–31]. Our results suggest that mutations in the PIK3CA-PTEN-AKT branch of the EGFR pathway have a lesser impact on malignant progression in CRC than mutations in the RAS-RAF-MAPK branch. It is also noteworthy that the majority of *PIK3CA* mutations in this study were found in exon 9, while previous studies have suggested that *PIK3CA* exon 20, but not *PIK3CA* exon 9, mutations are associated with cetuximab resistance in CRC [22]. Although the association between *PIK3CA* mutations and treatment resistance was not statistically significant, individual *PIK3CA* mutations tended to be associated with poorer clinical outcomes as indicated by increased SD or marginal PR. In contrast, tumors with concomitant mutations in *PIK3CA* and *KRAS* or in *PIK3CA* and *BRAF* were more resistant to cetuximab therapy as indicated by increased PD.

*PTEN* mutations are found in normal and neoplastic cells, and the significance of *PTEN* mutations in carcinogenesis is also unclear [32]. Several immunohistochemistry-based studies have linked loss of *PTEN* expression to the anti-tumor activity of cetuximab in advanced CRC [33–34]. However, we detected *PTEN* mutations in a small number of responders, suggesting either that *PTEN* mutations are poor predictors of response to cetuximab or that different experimental methods are needed when using *PTEN* as a biomarker in clinical routine diagnosis. The *PTEN* Y176C mutation only modestly decreased its catalytic activity and conformational stability [35], which could explain why carriers of this mutation still benefit from anti-EGFR therapy. The *AKT* E17K mutation has been linked to prolonged activation of the gene, which contributes to tumorigenesis by over-activating the mTOR pathway [36]. However, because the one non-responsive subject with an *AKT* mutation also carried a *BRAF* mutation, it was difficult for us to examine the individual predictive value of *AKT* for anti-EGFR treatment. Taken together, although our data indicate that mutations in *PIK3CA*, *PTEN* and *AKT* do not predict anti-EGFR therapy efficacy, more studies are needed to further evaluate these genes' clinical significance.

Because no mutations in any of the genes examined here were detected in 10 non-responsive subjects, it is likely that other carcinogenesis-associated genes also influence resistance to cetuximab treatment. Other factors, including increases in *EGFR* or *ALK* gene copy numbers, *MET* overexpression, and elevated *KRAS*, *MET*, and *ERBB* levels following cetuximab therapy, have been linked to CRC progression and deserve further investigation [37–39]. Finally, only surgically removed pre-treatment primary tumor tissues were analyzed in the current study. Previous studies have found some differences in *KRAS* mutation status between primary tumors and corresponding metastatic lesions [40–41]. Such genetic heterogeneity may explain why no additional mutations were detected in the non-responders.

In conclusion, we provide evidence that the detection of EGFR-pathway variants aids in the identification of EGFR therapy non-responders. Our data strongly suggest that mutation screening should be extended beyond *KRAS* exon 2 to include *KRAS* exons 3 and 4, *BRAF*, and *NRAS*. These variants were associated with poor tumor responses and shorter PFS in mCRC patients with wild-type *KRAS* who were treated with the anti-EGFR agent cetuximab.

## METHODS

### Patients

Fifty-three consecutive patients with mCRC who were treated between 2010 and 2014 at the Oncology Department of the Chang Gung Memorial Hospital, Tao-Yuan, Taiwan, were included in this study (see Table 1 for an overview of patient characteristics). Inclusion criteria were: (1) histologically confirmed colorectal adenocarcinoma, (2) metachronous or synchronous metastatic disease, (3) wild-type *KRAS* exon 2 in primary colorectal or metastatic tumors (confirmed by direct sequencing performed in the Pathology Department at Chang Gung, detection limit: 20%), (iv) previous combined cetuximab and oxaliplatin- or irinotecan-based chemotherapy treatment, (v) measurable radiological lesions, and (vi) good performance status (ECOG: 0–1). The study was approved by the Institutional Review Board at the Chang Gung Memorial Hospital (IRB 102–2850A3). Written informed consent was obtained from all patients before sample collection.

### Treatment regimens

In the first-line therapy group, patients with mCRC received cetuximab plus chemotherapy with FOLFIRI (irinotecan and infusional 5-fluorouracil with leucovorin) [8]. In the third-line group, patients with mCRC who experienced progression after irinotecan and oxaliplatin-based chemotherapy received cetuximab plus chemotherapy with IFL (irinotecan and bolus 5-fluorouracil with leucovorin) [42–43]. The detailed chemotherapy and cetuximab schedules were as follows: (i) IFL (Leucovorin 20 mg/m^2^ iv bolus qw x 4 weeks every 6 weeks, 5-FU 500 mg/m^2^ iv bolus qw x 4 weeks every 6 weeks, irinotecan 125 mg/m^2^ iv qw x 4 weeks every 6 weeks for 4 cycles) [42]; (ii) FOLFIRI regimen (Leucovorin 400 mg/m^2^ iv over 2 hours before 5-FU d1, 5-FU 400 mg/m^2^ iv bolus d1, and then 2400 mg/m^2^ iv over 46 hours, irinotecan 180 mg/m^2^ iv over 90 min d1 every 2 weeks for 12 cycles) [45]. (iii). Cetuximab infusion (with a loading dose of 400 mg/m^2^ and subsequent maintenance doses of 250 mg/m^2^/per week or 500 mg/m^2^ biweekly) [42–44, 46–47]. Toxicity was evaluated using Common Terminology Criteria (CTCAE) for Adverse Events, version 4.0 [48].

### Sample preparation, DNA sequencing, and data processing

Surgically removed pre-treatment primary tumor tissue specimens were fixed in formalin and preserved in paraffin blocks for histological examination and for prolonged storage. Two 10 μm-thick formalin-fixed paraffin-embedded (FFPE) sections were used for this study. Genomic DNA was extracted from FFPE tumor samples using the QIAamp DNA FFPE Tissue Kit (Qiagen). DNA was quantified using the Quant-iT dsDNA HS Assay (Invitrogen). The integrity of genomic DNA was determined using the Fragment Analyzer assay (Advanced Analytical Technologies, Inc.) and quantitative PCR.

Twenty ng of genomic DNA were amplified using a pool of primers to enrich frequently mutated hotspot regions of 10 genes that are related to the EGFR pathway (*EGFR, KRAS, HRAS, NRAS, BRAF, PIK3CA, AKT1, PTEN, HER2*, and *HER4*) and *TP53*. Complete targeted regions and primer sequences are listed in Supplementary Table 2. The entire sequencing region covered 6225 bases and included 2611 entries from the Catalogue of Somatic Mutation in Cancer (COSMIC) database (version 68). Amplicons were ligated with barcoded adaptors using the Ion Amplicon Library Kit (Life Technologies). Barcoded libraries were subsequently conjugated with sequencing beads by emulsion PCR and enriched using Ion OneTouch2 and OneTouch ES (Life Technologies) according to the manufacturer's protocol. Sequencing was performed using the Ion Torrent PGM system with the Ion 318 chip. Raw reads were mapped to the hg19 reference genome using Torrent Suite Server (version 4.2) and variants were identified using the Torrent Variant Caller plug-in (version 4.2). Per the manufacturer's recommendation, the variant calling threshold was set at 5% for variants without COSMIC IDs and 2% for variants with COSMIC IDs. Variants were annotated using the Variant Effect Predictor (VEP, version 78). Common variants (MAF ≥ 1%) in the dbSNP database (build 138) or in the 1000 Genome project (phase 1) without COSMIC entries were filtered out. Only non-synonymous mutations were analyzed.

### Statistical analysis

Efficacy of anti-EGFR therapy was evaluated by a CT scan every 3 months according to RECIST criteria version 1.1 [49]. Patients were categorized as responders if they achieved complete response (CR) or partial response (PR) and non-responders if they demonstrated stable disease (SD) or progressive disease (PD). Progression-free survival (PFS) was calculated from the first day of therapy to the date of proven progression, or death. The last date when the patient was seen alive without recurrence was used for those who could not be located for follow-up.

The significance of the association between individual clinical factors was evaluated using Fisher's exact test. The survival rate was calculated by the Kaplan-Meier method and the statistical significance of the differences was evaluated using the log-rank test. All statistical tests were two-sided, and *p* < 0.05 was considered statistically significant. All statistical analyses were performed using SPSS version 18.0 for Windows (SPSS Inc., Chicago, IL., USA).

## SUPPLEMENTARY MATERIALS FIGURE AND TABLES


