# Supplementary material for: Mutations of KRAS/NRAS/BRAF predict cetuximab resistance in metastatic colorectal cancer patients
Source: Oncotarget. 2016 Mar 12;7(16):22257–70. doi: 10.18632/oncotarget.8076 (PMC5008360; doi:10.18632/oncotarget.8076)
Supplement: Supplementary file 1 [file oncotarget-07-22257-s001.pdf]

Mutations of *KRAS/NRAS/BRAF* predict cetuximab resistance in metastatic colorectal cancer patients

Supplementary Materials

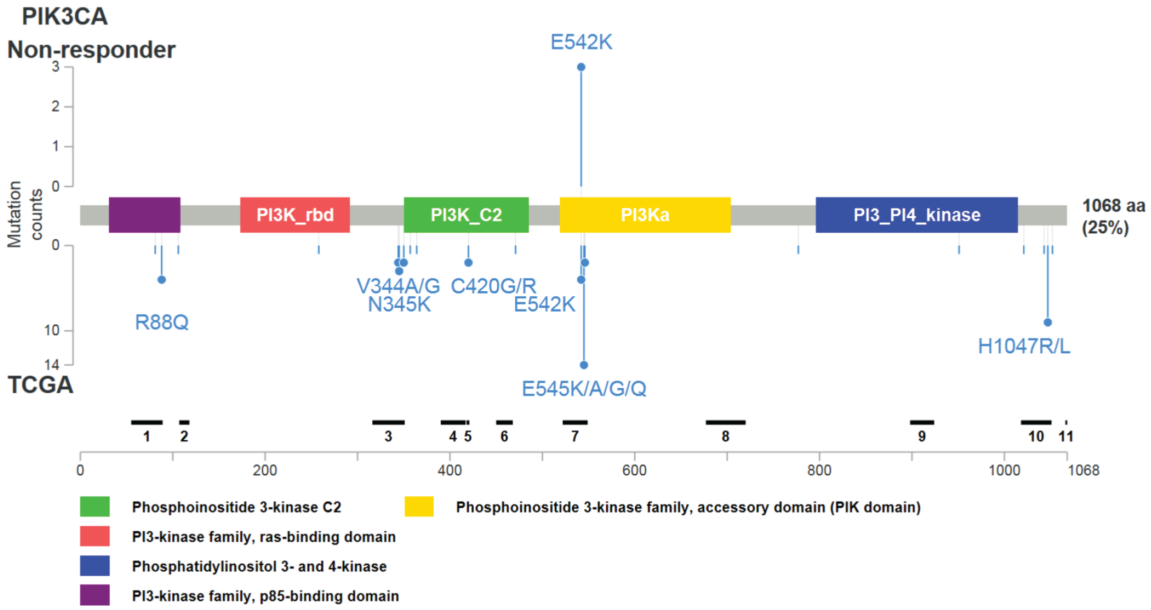

Supplementary Figure S1: Distribution of *PIK3CA* genetic alterations detected in the non-responder group. Schematic representation of the protein structure of *PIK3CA*. The upper panel indicates the genetic alterations detected in the non-responder group. The lower panel indicates the mutation spectrum in The Cancer Genome Atlas (TCGA) colorectal cancer dataset. The left scale indicates the number of cases. The blue, yellow, red, and purple circles indicate missense mutations, insertions/deletions, nonsense mutations, and multiple types of mutations, respectively.

Supplementary Table S1: Genomic regions sequenced

Gene name	chromosome	Amplicon start	Amplicon end	Transcript ID	Exon	Exon length	Exon start	Exon end
AKT1	chr14	105241434	105241519	ENST00000554581	5	132	105241413	105241544
AKT1	chr14	105246446	105246583	ENST00000554581	2	129	105246425	105246553
BRAF	chr7	140453103	140453221	ENST00000288602	15	119	140453075	140453193
BRAF	chr7	140481392	140481515	ENST00000288602	11	118	140481376	140481493
EGFR	chr7	55211045	55211126	ENST00000275493	3	184	55210998	55211181
EGFR	chr7	55221793	55221919	ENST00000275493	7	142	55221704	55221845
EGFR	chr7	55232963	55233053	ENST00000275493	15	158	55232973	55233130
EGFR	chr7	55241636	55241729	ENST00000275493	18	123	55241614	55241736
EGFR	chr7	55242412	55242540	ENST00000275493	19	99	55242415	55242513
EGFR	chr7	55248966	55249090	ENST00000275493	20	186	55248986	55249171
EGFR	chr7	55249123	55249245	ENST00000275493	20	186	55248986	55249171
EGFR	chr7	55259508	55259628	ENST00000275493	21	156	55259412	55259567
ERBB2	chr17	37880213	37880340	ENST00000269571	19	99	37880165	37880263
ERBB2	chr17	37880954	37881061	ENST00000269571	20	186	37880979	37881164
ERBB2	chr17	37881325	37881453	ENST00000269571	21	156	37881302	37881457
ERBB4	chr2	212288905	212288990	ENST00000342788	23	147	212288880	212289026
ERBB4	chr2	212530052	212530180	ENST00000342788	15	155	212530048	212530202
ERBB4	chr2	212576800	212576910	ENST00000342788	9	127	212576775	212576901
ERBB4	chr2	212578289	212578415	ENST00000342788	8	114	212578260	212578373
ERBB4	chr2	212587134	212587239	ENST00000342788	7	142	212587118	212587259
ERBB4	chr2	212589765	212589867	ENST00000342788	6	119	212589801	212589919
ERBB4	chr2	212652720	212652806	ENST00000342788	4	135	212652750	212652884
ERBB4	chr2	212812076	212812169	ENST00000342788	3	187	212812155	212812341
HRAS	chr11	533813	533930	ENST00000451590	3	179	533766	533944
HRAS	chr11	534221	534308	ENST00000451590	2	164	534212	534375
KRAS	chr12	25378550	25378658	ENST00000311936	4	160	25378548	25378707
KRAS	chr12	25380261	25380364	ENST00000311936	3	179	25380168	25380346
KRAS	chr12	25398187	25398304	ENST00000311936	2	122	25398208	25398329
NRAS	chr1	115252186	115252269	ENST00000369535	4	160	115252190	115252349
NRAS	chr1	115256505	115256584	ENST00000369535	3	179	115256421	115256599
NRAS	chr1	115258690	115258774	ENST00000369535	2	128	115258671	115258798
PIK3CA	chr3	178916776	178916881	ENST00000263967	2	428	178916538	178916965
PIK3CA	chr3	178916932	178917035	ENST00000263967	2	428	178916538	178916965
PIK3CA	chr3	178921465	178921570	ENST00000263967	5	246	178921332	178921577
PIK3CA	chr3	178927406	178927525	ENST00000263967	7	106	178927383	178927488
PIK3CA	chr3	178927902	178927986	ENST00000263967	8	153	178927974	178928126
PIK3CA	chr3	178928070	178928160	ENST00000263967	8	153	178927974	178928126

PIK3CA	chr3	178936024	178936105	ENST00000263967	10	125	178935998	178936122
PIK3CA	chr3	178938788	178938918	ENST00000263967	14	172	178938774	178938945
PIK3CA	chr3	178947819	178947896	ENST00000263967	19	118	178947792	178947909
PIK3CA	chr3	178951997	178952097	ENST00000263967	21	6000	178951882	178957881
PIK3CA	chr3	178952141	178952237	ENST00000263967	21	6000	178951882	178957881
PTEN	chr10	89624208	89624300	ENST00000371953	1	1436	89622870	89624305
PTEN	chr10	89685259	89685374	ENST00000371953	3	45	89685270	89685314
PTEN	chr10	89692814	89692920	ENST00000371953	5	239	89692770	89693008
PTEN	chr10	89711805	89711932	ENST00000371953	6	142	89711875	89712016
PTEN	chr10	89717504	89717620	ENST00000371953	7	167	89717610	89717776
PTEN	chr10	89717667	89717780	ENST00000371953	7	167	89717610	89717776
PTEN	chr10	89720696	89720747	ENST00000371953	8	225	89720651	89720875
PTEN	chr10	89720785	89720900	ENST00000371953	8	225	89720651	89720875
TP53	chr17	7573924	7574035	ENST00000269305	10	107	7573927	7574033
TP53	chr17	7577016	7577151	ENST00000269305	8	137	7577019	7577155
TP53	chr17	7577509	7577612	ENST00000269305	7	110	7577499	7577608
TP53	chr17	7578181	7578298	ENST00000269305	6	113	7578177	7578289
TP53	chr17	7578353	7578483	ENST00000269305	5	184	7578371	7578554
TP53	chr17	7578517	7578601	ENST00000269305	5	184	7578371	7578554
TP53	chr17	7579351	7579485	ENST00000269305	4	279	7579312	7579590
TP53	chr17	7579854	7579960	ENST00000269305	2	102	7579839	7579940

Supplementary Table S2: Run stats

Sample ID	Total read	On target reads	Mean depth	Uniformity
A00006	925297	97%	4007	99%
A00007	817417	95%	3489	98%
A00008	2583251	98%	11060	95%
A00009	2379819	97%	10193	94%
A00011	2685576	98%	11740	90%
A00013	3508536	98%	15564	94%
A00014	3025868	98%	13452	97%
A00015	624371	98%	2625	90%
A00018	1022074	96%	4469	97%
A00019	969377	97%	4232	97%
A00020	842484	96%	3622	98%
A00021	1046358	97%	4522	94%
A00022	869059	96%	3755	96%
A00023	1693608	94%	6595	79%
A00024	1005872	98%	4444	96%
A00025	919478	96%	4041	97%
A00026	1000694	98%	4332	95%
A00027	827929	98%	3612	97%
A00029	16351460	97%	69863	91%
A00032	4676967	95%	20108	96%
A00035	3488703	96%	15075	91%
A00037	4001072	98%	17716	94%
A00041	1717151	90%	6342	78%
A00046	744878	96%	3318	96%
A00047	1114174	97%	4902	97%
A00051	1132020	97%	4971	96%
A00055	2607003	97%	10809	80%
A00074	2197841	99%	9803	93%
A00075	2038598	98%	9091	96%
A00076	2219380	98%	9752	97%
A00078	2503603	99%	11168	98%
A00079	2016868	97%	8894	97%
A00080	3443267	99%	15403	91%
A00082	4664267	98%	20638	96%
A00083	2028064	98%	9024	98%
A00084	2529958	99%	11428	88%
A00085	2362093	98%	10478	96%
A00086	2599838	94%	11056	97%

A00087	2590460	98%	11558	90%
A00089	2720412	99%	12064	90%
A00090	2807721	99%	12574	96%
A00091	2281861	96%	10013	95%
A00092	2712699	98%	12014	96%
A00170	1475920	98%	6295	94%
A00172	807488	98%	3475	99%
A00173	667340	97%	2794	94%
A00178	398841	97%	1656	95%
A00181	771966	98%	3408	97%
A00184	736618	97%	3131	90%
A00185	810426	95%	3405	91%
A00187	821805	98%	3567	93%
A00188	733742	96%	3062	95%
A00189	540782	97%	2343	90%

Supplementary Table S3: List of mutations

Gene	Exon	cds change	aa change	mutation type	Chr	Position	NM_ID	Reference	Variant	Variant_ frequency	Varaint_ counts	Reference_ counts	Total_ depth	Sample
AKT1	2	c.49G > A	p.E17K	missense, splice_ region	chr14	105246551	NM_005163	C	T	27.9%	546	1414	1960	A00021
BRAF	11	c.1397G > C	p.G466A	missense	chr7	140481411	NM_004333	C	G	38.1%	761	1235	1996	A00085
BRAF	11	c.1406G > C	p.G469A	missense	chr7	140481402	NM_004333	C	G	19.7%	388	1577	1965	A00021
BRAF	11	c.1406G > C	p.G469A	missense	chr7	140481402	NM_004333	C	G	28.1%	557	1425	1982	A00082
BRAF	15	c.1781A > G	p.D594G	missense	chr7	140453154	NM_004333	T	C	26.7%	300	825	1125	A00026
BRAF	15	c.1799T > A	p.V600E	missense	chr7	140453136	NM_004333	A	T	32.4%	641	1339	1980	A00083
BRAF	15	c.1799T > A	p.V600E	missense	chr7	140453136	NM_004333	A	T	17.8%	353	1630	1984	A00091
KRAS	3	c.182A > T	p.Q61L	missense	chr12	25380276	NM_004985	T	A	9.3%	186	1812	1998	A00032
KRAS	3	c.182A > T	p.Q61L	missense	chr12	25380276	NM_004985	T	A	54.5%	1085	907	1992	A00185
KRAS	3	c.183A > C	p.Q61H	missense	chr12	25380275	NM_004985	T	G	36.8%	733	1261	1994	A00187
KRAS	4	c.436G > A	p.A146T	missense	chr12	25378562	NM_004985	C	T	18.2%	363	1636	1999	A00013
KRAS	4	c.436G > A	p.A146T	missense	chr12	25378562	NM_004985	C	T	10.7%	92	769	861	A00178
NRAS	2	c.34G > T	p.G12C	missense	chr1	115258748	NM_002524	C	A	6.8%	136	1853	1989	A00080
NRAS	2	c.35G > C	p.G12A	missense	chr1	115258747	NM_002524	C	G	23.4%	466	1529	1995	A00011
NRAS	2	c.38G > A	p.G13D	missense	chr1	115258744	NM_002524	C	T	41.7%	834	1166	2000	A00085
NRAS	3	c.181C > A	p.Q61K	missense	chr1	115256530	NM_002524	G	T	47.1%	931	1044	1975	A00188
PIK3CA	10	c.1624G > A	p.E542K	missense	chr3	178936082	NM_006218	G	A	15.6%	291	1571	1862	A00026
PIK3CA	10	c.1624G > A	p.E542K	missense	chr3	178936082	NM_006218	G	A	22.3%	439	1526	1965	A00090
PIK3CA	10	c.1624G > A	p.E542K	missense	chr3	178936082	NM_006218	G	A	31.0%	612	1362	1974	A00172
PIK3CA	10	c.1624G > A	p.E542K	missense	chr3	178936082	NM_006218	G	A	31.0%	608	1356	1964	A00187
PIK3CA	10	c.1633G > A	p.E545K	missense	chr3	178936091	NM_006218	G	A	7.4%	147	1837	1984	A00041
PIK3CA	21	c.3136G > A	p.A1046T	missense	chr3	178952081	NM_006218	G	A	16.3%	323	1659	1982	A00076
PTEN	-	c.209+5G > A	-	splice_ region	chr10	89685319	NM_000314	G	A	43.2%	858	1127	1985	A00078
PTEN	6	c.527A > G	p.Y176C	missense	chr10	89711909	NM_000314	A	G	52.3%	1007	917	1924	A00046
TP53	5	c.451C > A	p.P151T	missense	chr17	7578479	NM_000546	G	T	34.4%	686	1309	1995	A00046
TP53	5	c.481G > A	p.A161T	missense	chr17	7578449	NM_000546	C	T	42.4%	819	1114	1933	A00027
TP53	5	c.488A > G	p.Y163C	missense	chr17	7578442	NM_000546	T	C	18.6%	369	1615	1984	A00024
TP53	5	c.503A > G	p.H168R	missense	chr17	7578427	NM_000546	T	C	5.0%	42	790	832	A00041
TP53	5	c.517G > A	p.V173M	missense	chr17	7578413	NM_000546	C	T	42.7%	851	1029	1995	A00022
TP53	5	c.517G > C	p.V173L	missense	chr17	7578413	NM_000546	C	G	5.8%	115	1029	1995	A00022
TP53	5	c.524G > A	p.R175H	missense	chr17	7578406	NM_000546	C	T	32.6%	652	1345	1997	A00013
TP53	5	c.524G > A	p.R175H	missense	chr17	7578406	NM_000546	C	T	35.4%	705	1287	1992	A00046
TP53	5	c.524G > A	p.R175H	missense	chr17	7578406	NM_000546	C	T	24.5%	489	1508	1997	A00076

TP53	5	c.524G > A	p.R175H	missense	chr17	7578406	NM_000546	C	T	46.5%	928	1067	1996	A00085
TP53	5	c.524G > A	p.R175H	missense	chr17	7578406	NM_000546	C	T	23.0%	459	1536	1995	A00091
TP53	5	c.524G > A	p.R175H	missense	chr17	7578406	NM_000546	C	T	72.7%	1277	479	1756	A00172
TP53	5	c.527G > A	p.C176Y	missense	chr17	7578403	NM_000546	C	T	67.3%	1334	633	1983	A00090
TP53	5	c.528C > A	p.C176*	nonsense	chr17	7578402	NM_000546	G	T	21.3%	424	1568	1992	A00021
TP53	5	c.537T > G	p.H179Q	missense	chr17	7578393	NM_000546	A	C	10.7%	204	1698	1902	A00087
TP53	6	c.574C > T	p.Q192*	nonsense	chr17	7578275	NM_000546	G	A	63.5%	1146	659	1805	A00023
TP53	6	c.577C > T	p.H193Y	missense	chr17	7578272	NM_000546	G	A	20.7%	376	1442	1818	A00025
TP53	6	c.577C > T	p.H193Y	missense	chr17	7578272	NM_000546	G	A	52.5%	949	857	1806	A00078
TP53	6	c.580C > T	p.L194F	missense	chr17	7578269	NM_000546	G	A	9.7%	171	1592	1763	A00024
TP53	6	c.586C > T	p.R196*	nonsense	chr17	7578263	NM_000546	G	A	65.4%	1299	687	1986	A00037
TP53	6	c.610G > T	p.E204*	nonsense	chr17	7578239	NM_000546	C	A	11.2%	203	1614	1817	A00178
TP53	6	c.637C > T	p.R213*	nonsense	chr17	7578212	NM_000546	G	A	9.1%	178	1770	1948	A00181
TP53	6	c.637C > T	p.R213*	nonsense	chr17	7578212	NM_000546	G	A	81.3%	1365	314	1679	A00188
TP53	7	c.714 dupT	p.N239*	frameshift	chr17	7577566	NM_000546	T	TA	11.2%	222	1767	1989	A00055
TP53	7	c.743G > A	p.R248Q	missense	chr17	7577538	NM_000546	C	T	42.9%	835	1113	1948	A00170
TP53	8	c.797G > A	p.G266E	missense	chr17	7577141	NM_000546	C	T	70.5%	1410	589	1999	A00084
TP53	8	c.817C > T	p.R273C	missense	chr17	7577121	NM_000546	G	A	40.3%	798	1184	1982	A00020
TP53	8	c.818G > A	p.R273H	missense	chr17	7577120	NM_000546	C	T	33.8%	668	1309	1977	A00074
TP53	8	c.818G > A	p.R273H	missense	chr17	7577120	NM_000546	C	T	51.6%	731	686	1417	A00185
TP53	8	c.820G > T	p.V274F	missense	chr17	7577118	NM_000546	C	A	34.6%	689	1300	1989	A00019
TP53	8	c.844C > T	p.R282W	missense	chr17	7577094	NM_000546	G	A	12.8%	247	1688	1935	A00089
TP53	8	c.845G > C	p.R282P	missense	chr17	7577093	NM_000546	C	G	19.3%	385	1610	1995	A00024
TP53	8	c.916C > T	p.R306*	nonsense	chr17	7577022	NM_000546	G	A	67.6%	1351	647	1998	A00035
TP53	8	c.916C > T	p.R306*	nonsense	chr17	7577022	NM_000546	G	A	38.9%	307	483	790	A00184
TP53	10	c.1024C > T	p.R342*	nonsense	chr17	7574003	NM_000546	G	A	27.7%	553	1447	2000	A00007
TP53	10	c.1024C > T	p.R342*	nonsense	chr17	7574003	NM_000546	G	A	30.1%	602	1397	1999	A00075
TP53	10	c.1024C > T	p.R342*	nonsense	chr17	7574003	NM_000546	G	A	42.4%	366	497	863	A00189
